# Immunopathogenesis of multiple sclerosis

**DOI:** 10.4103/0972-2327.58274

**Published:** 2009

**Authors:** Michael K. Racke

**Affiliations:** The Helen C. Kurtz Chair of Neurology, The Ohio State University Medical Center, 395 West 12^th^ Avenue, Columbus, OH 43210 USA

**Keywords:** Magnetic resonance imaging, multiple sclerosis, pathogenesis, review

## Abstract

Multiple sclerosis (MS) is a suspected autoimmune disease in which myelin-specific CD4+ and CD8+ T cells enter the central nervous system (CNS) and initiate an inflammatory response directed against myelin and other components of the CNS. Acute MS exacerbations are believed be the result of active inflammation, and progression of disability is generally believed to reflect accumulation of damage to the CNS, particularly axonal damage. Over the last several years, the pathophysiology of MS is being appreciated to be much more complex, and it appears that the development of the MS plaque involves a large number of cell populations, including CD8+ T lymphocytes, B cells, and Th17 cells (a population of helper T cells that secrete the inflammatory cytokine IL-17). The axonal transection and degeneration that is thought to represent the basis for progressive MS is now recognized to begin early in the disease process and to continue in the progressive forms of the disease. Molecules important for limiting aberrant neural connections in the CNS have been identified, which suppress axonal sprouting and regeneration of transected axons within the CNS. Pathways have also been identified that prevent remyelination of the MS lesion by oligodendrocyte precursors. Novel neuroimaging methodologies and potential biomarkers are being developed to monitor various aspects of the disease process in MS. As we identify the pathways responsible for the clinical phenomena of MS, we will be able to develop new therapeutic strategies for this disabling illness of young adults.

Multiple sclerosis (MS) is a disorder of the central nervous system (CNS) with features of inflammation, demyelination, and neurodegeneration.[1] Exacerbations of MS are believed to reflect inflammatory episodes, while the neurodegenerative aspects of gliosis and axonal loss result in the progression of disability.[2] Recent studies in MS have identified new cellular and cytokine pathways involved in disease pathogenesis. In addition, advances in the imaging of MS have added new insights into the pathogenesis of the disease and provided additional methods for monitoring the disease process.

## Pathogenesis of MS: Inflammation, Demyelination, and Neurodegeneration

Over the years, MS has been considered to be an autoimmune disorder where myelin-specific T cells initiate an inflammatory process that results in CNS demyelination.[1] These autoreactive T cells are believed to become activated in the periphery and to upregulate adhesion molecules that allow these T cells to interact with and cross the blood brain–barrier and finally establish an inflammatory response directed against myelin.[3] It is well known that healthy individuals have myelin-reactive T cells; however, they are typically of a naïve phenotype and do not migrate into the CNS and establish an inflammatory response.[4] In contrast, patients with MS have been observed to have myelin-specific T cells which are of an activated or memory phenotype and are more likely to be of a Th1 phenotype.[1,5] Exactly how these autoreactive, myelin-specific T cells become activated is still a matter of active investigation. Processes such as molecular mimicry, where T cells respond to environmental antigens that resemble self-antigens, could be a potential mechanism by which these cells get activated.[4,6] Although MS has been traditionally considered a disease of the white matter, attention is now also being paid to lesions in the gray matter and cortex.[7]

In recent years, MS research has focused on the role of CD4+ T cells in disease pathogenesis. Much of this is due to similarities between MS and its animal model (experimental autoimmune encephalomyelitis; EAE), which is typically induced by CD4+ T cells.[1,8] Several recent studies have begun to swing the pendulum with regard to the study of other immune cell populations in MS. The role of CD8+ T cells has received additional attention because they are prominent in the inflammatory infiltrate in MS lesions, have been described to recognize myelin antigens in MS patients, and play a role in breakdown of the blood–brain barrier.[8,9] The role of B cells is MS pathogenesis has also received increased attention.[6] B cells may secrete antibodies that recognize and participate in myelin breakdown.[6] The potential importance of B cells in the pathogenesis of MS has been highlighted by studies utilizing the monoclonal antibody rituximab, which recognizes CD20 on B cells and thus results in their depletion. In addition, studies on rituximab have suggested significant reduction in relapses in patients with relapsing remitting MS (RRMS).[10] There are also studies which now suggest that MS may initially arise as a result of a primary insult to oligodendrocytes and that the immune-mediated inflammatory process is a secondary phenomenon.[11] Interestingly, studies examining genetic susceptibility in MS continue to identify immune-related genes that confer increased susceptibility, with genes for the IL-7R and IL-2Ralpha chain, as well as the MHC locus, being recognized as conferring increased risk.[12] These studies argue that the immune system plays a major role in the initiation of MS, although other factors could play a significant role in modifying disease outcome.

There was a time when the accumulation of disability in MS was thought to be the result of chronic demyelination. More recently, it is generally accepted that progression of disability in MS is more likely related to axonal dysfunction, including axonal transection and Wallerian degeneration.[13,14] Axonal loss within MS lesions may vary, with these areas having 20–80% of the axon density seen in adjacent normal-appearing white matter,[2] and the degree of loss is significantly related to disease progression.[15] Axonal transection in MS can occur through several mechanisms.[2] Inflammatory mediators released as part of the immune activation occurring in the MS lesion may damage axons. Axonal injury may also occur in the chronic demyelinated plaque as the result of lack of neurotrophic factors being provided to the axon by oligodendrocytes. While it is logical to consider that axonal injury is responsible for the transition from relapsing–remitting MS to progressive forms of the disease, it should be noted that axonal transection is observed early in the course of the disease, and is even present in patients at the earliest stages of the disease (clinically isolated syndromes; CIS).[13,16] Patients in the early stages of MS appear to tolerate this axonal loss because the remaining tissue is able to compensate for the initial injury.[13] The process of early axonal loss as a consequence of inflammatory demyelination suggests that strategies that prevent inflammation early in the disease process may be very influential in altering the pathogenic process in MS.[3,14] In addition, processes have been identified that suppress neurite sprouting and the regrowth of transected axons in the adult mammalian CNS. For example Nogo, a protein produced by oligodendrocytes, interacts with the Nogo receptor present on axons to inhibit neurite outgrowth.[17] Since the identification of Nogo several other molecules in myelin have been identified that suppress neurite sprouting in the adult CNS, including myelin-associated glycoprotein (MAG) and oligodendrocyte myelin glycoprotein (OMgp), both of which also stimulate Nogo receptors.[17,18] The suppression of neurite sprouting by Nogo and related molecules is likely a mechanism that prevents the formation of aberrant connections within the brain. In MS, processes such as Nogo receptor stimulation prevent transected axons from regrowth and repair with demyelinated tissue. Recovery from an MS exacerbation likely does not reflect remyelination but, rather, is the result of the re-establishment of transmission along the demyelinated axons by redistribution of sodium channels, permitting some degree of restoration of neuronal conduction.[14]

Another area of research has focused on the role of astrocytes in the pathogenesis of MS. Because oligodendrocytes have been known to provide trophic support to axons, the loss of oligodendrocytes has indirectly contributed to axonal degeneration.[3] Another feature of the MS plaque is its well-defined border, which has been identified as a barrier to penetration by oligodendrocyte precursor cells.[1] Because only a few oligodendrocyte precursors are able to enter the MS plaque, effective remyelination is successful in only a small number of lesions.[19,20] Some studies have suggested that astrocytes may contribute to the limited remyelination in the MS lesion. Production of transforming growth factor-β (TGFβ) by astrocytes results in increased expression of the molecule Jagged1, which interacts with the Notch1 receptor on the oligodendrocyte cell surface.[20] Activation of the Notch1 receptors inhibits oligodendrocyte differentiation and reduces subsequent remyelination.[20] The environment of an astroglial scar prevents oligodendrocyte precursors from entering and remyelinating the MS plaque, resulting in the loss of trophic support for neurons in the plaque and subsequent axonal degeneration. It has also been suggested that in addition to microglia, astrocytes may participate as antigen-presenting cells in activating self-reactive T lymphocytes.[4] Astrocytes can also express adhesion molecules that facilitate the entry of T lymphocytes into the CNS and, by releasing matrix metalloproteases, can participate in the breakdown of tight junctions between vascular endothelial cells.[21]

**Figure 1:**
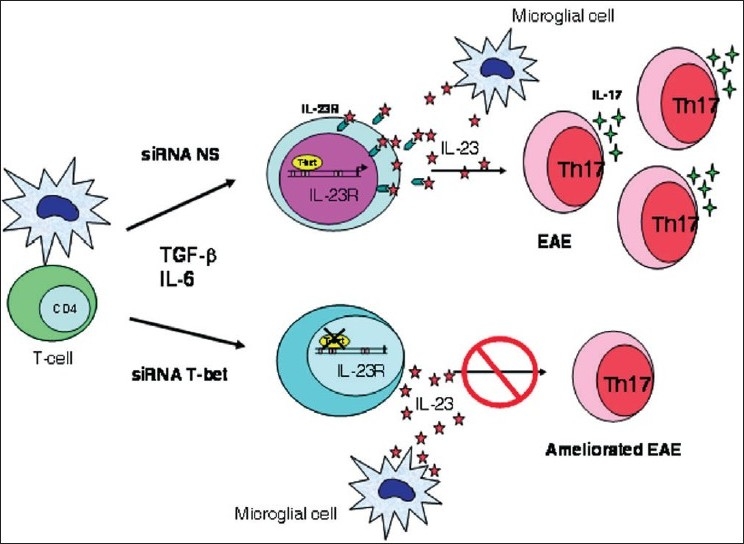
Th17 cells are present in the CNS and expand in the presence of IL-23. Resident CNS microglial cells have the capacity to produce IL-23, IL-6, and TGF-β, which could contribute to the differentiation and expansion of this unique Th17 cell population observed in the CNS. Silencing T-bet with siRNA inhibits IL-23R expression and subsequent expansion of Th17 cells.[24]

Finally, a recent area of active research has focused on cytokines that participate in effector mechanisms of T cells in EAE and MS. Cytokines of the IL-12/IL-23 family appear to be very important in the development of autoimmune diseases, including MS. IL-23 is required for the development of EAE and it stimulates the proliferation of a population of T cells that secrete IL-17 (Th17 cells); many investigators believe that these cells are crucial in the pathogenesis of MS. IL-23 and IL-17 are both expressed in MS lesions,[22] and increased expansion of Th17 cells is believed to contribute to the infiltration of myelin-specific T cells into the CNS [Figure 1].[23] In EAE model, the suppression of Th17 lymphocytes has been shown to reduce the clinical expression of disease, although this is becoming an area of controversy.[24,25]

## Neuroimaging Techniques in MS

MRI has become a useful tool for the diagnosis of MS and in the evaluation of the effects of treatment, particularly as an outcome measure in clinical trials. MRI is also helpful in determining the likelihood of conversion to clinically definite MS in patients with CIS.[26] T2 hyperintense lesions are nonspecific indicators of tissue injury that may reflect a number of disease processes, including edema, astrogliosis, axonal injury, demyelination, and remyelination.[27] T2 MRI provides little specific information about the various pathologic processes occurring within the CNS and is only weakly predictive of the future course of illness.[27–29]

Other imaging techniques provide more specific information about the pathologic processes that occur during MS.[27] Gadolinium (Gd)-enhancing lesions result from the leakage of this paramagnetic material across the blood–brain barrier, which is caused by the migration of inflammatory cells into the CNS. Gd-enhancing lesions indicate regions of active inflammation and breakdown of the blood–brain barrier. Chronic T1 hypointensities (T1 black holes) are a more specific measure of axonal loss or edema, and correlate more closely with disease progression than T2 lesion burden.[27,28] Magnetization transfer MRI (MT MRI) is a technique that examines the interactions of protons in free fluid and protons that are bound to macromolecules.[30] Several studies have demonstrated that MT MRI is able to detect neuronal injury in normal-appearing gray and white matter and that changes in MT MRI often precede macroscopic lesion formation.[30] Diffusion tensor MRI (DT MRI) distinguishes tissue based on the diffusion of water molecules.[31] In patients with MS, changes in water diffusivity on DT MRI have been associated with demyelination and axonal loss in lesions present on T1 or T2 imaging and in normal-appearing white matter.[31] In patients with progressive MS, diffusion abnormalities identified using DT MRI have been predictive of the worsening of MS disability over time.[32] Proton magnetic resonance spectroscopy (MRS) uses the spectra of magnetic resonance signals to identify a number of specific chemical compounds, including choline, creatine, lactate, and n-acetylaspartate (NAA, a marker of axonal integrity), in MS lesions or normal-appearing white matter.[33] NAA in the adult brain is expressed almost exclusively in neurons and neuronal processes, and NAA concentrations have been used to determine axonal density and to demonstrate alterations in gray matter in patients with MS.[33,34] Elevated choline levels occur in acute MS lesions as a consequence of the release of membrane phospholipids during myelin degradation.[33] Optical coherence tomography (OCT) is another new technique used to evaluate the thickness of retinal nerve fiber layer in patients with glaucoma and other retinal diseases.[35] Optic neuritis in patients with MS is associated with decreased thickness of the retinal nerve fiber layer, which can be quantified using OCT. Macular volume loss has been demonstrated to be quite significant in patients with secondary progressive MS.[35] OCT has emerged as a potential marker for axonal injury in patients with MS, and retinal nerve fiber layer thickness has been shown to correlate strongly with brain atrophy and disability.[35,36]

Functional MRI (fMRI) is another technique which has been used to assess brain function during different tasks by quantifying regional differences in the concentration of deoxyhemoglobin, which reflects blood flow and oxygen consumption due to neural activity.[37] Studies using fMRI in patients with MS has shown the recruitment of brain areas that are not normally activated during a particular task.[37] For example, a simple hand movement will elicit widespread activation of sensory and motor cortical regions among patients with MS, though this is not seen in healthy subjects. This recruitment of additional brain regions not normally utilized in a task might provide an explanation for some of the fatigue that is experienced among patients with MS, as heightened brain activity may significantly increase total body energy expenditure. Another recent development has been the use of higher-field-strength magnets in the imaging of patients with MS. Conventional MRI using 1.5-T magnets detect only a small fraction of cortical gray matter lesions that are identified histopathologically.[38] Studies using MRI magnets with field strengths as high as 8 T have demonstrated that many more lesions are present in the cortex than can be appreciated with a typical 1.5-T magnet.[39] These powerful, high-field-strength magnets have made it possible to view MS lesions at very high levels of resolution. One insight from these studies is that almost all MS lesions surround a blood vessel, which provides additional support for the hypothesis that MS is mediated by an immune process. High-power magnets may help to define changes that occur within the CNS that currently are not appreciated with conventional MRI. For example, there have been several studies of the immunosuppressive agent cladribine, treatment with which produced a dramatic inhibition of MRI activity (identified using conventional MRI); however, these patients continued to exhibit clinical progression of MS. Studies that have used more powerful magnets have shown that there is probably much greater disease activity than was previously suspected, much of which was not apparent with conventional MRI imaging. In the BECOME clinical trial, which compared the efficacy of interferon beta-1b *vs* glatiramer acetate (GA), the primary outcome measure was disease activity as measured using MRI with 3-T magnets and triple-dose gadolinium.[40] The investigators expected to demonstrate the superiority of interferon beta-1b over GA on disease activity measured with MRI. However, imaging results obtained with the high-power magnet revealed no significant differences between the two treatments; increasing the sensitivity for disease activity may have eliminated any difference appreciated between the two agents.

## Biological Markers of MS

Reliable biological markers of MS disease activity could be useful for the diagnosis of MS, for assessing prognosis, and as a method for evaluating the effects of therapy. Cerebrospinal fluid (CSF) markers such as oligoclonal bands or IgG index are often present in patients with MS and have been used as markers for the disease, but these markers are also present in patients with other inflammatory CNS conditions.[41] Potential markers of greater sensitivity and specificity are being evaluated in MS clinical trials. As noted previously, the oligodendrocyte protein Nogo inhibits axonal sprouting and regrowth. Nogo A is found primarily in the CNS, including in oligodendrocytes and neurons; Nogo B is expressed throughout the body; and Nogo C is found primarily in muscle.[41] Detection of Nogo A in CSF has been proposed as a sensitive and specific biomarker for MS, including both relapsing and progressing forms of the disease. An examination of Nogo A in CSF found a soluble Nogo A fragment in 110 of 114 CSF samples from patients with MS (96%), but not in any of the samples obtained from more than 150 control subjects with other CNS disorders, including meningoencephalomyelitis and other CNS autoimmune disorders.[41]

Studies of the molecular changes that occur in MS also provide important information about the nature of the immune response in individuals with MS, and about how this response differs from healthy individuals. This information may provide additional approaches to selectively regulate immune function to induce a state that is more similar to that of healthy subjects. For example, a recent proteomic analysis of MS lesions was conducted to identify proteins unique to different types of MS lesions (acute plaques, chronic active plaques, and chronic plaques).[42] Surprisingly, this analysis identified abnormally expressed proteins that normally participate in the coagulation process (e.g., protein C and tissue factor) within MS lesions. Additional experiments demonstrated that that in addition to their effects on coagulation, these proteins also stimulated production of cytokines by Th1 and Th17 lymphocytes. Studies such as this one are important because they suggest that one can rationally develop treatments that are based on the molecular mechanisms of the disease. Natalizumab is currently the only medication that was developed rationally from preclinical models of disease to target a particular pathologic process that was believed to be important in MS. Initial studies identified α4 integrin as a an important factor in lymphocyte adhesion to cerebrovascular endothelium, and an antibody against α4 integrin was tested to see if it could inhibit that process. Subsequent preclinical and clinical research demonstrated that this antibody improved clinical outcomes in the EAE model and later this was also demonstrated in patients with MS.[43,44] Techniques such as proteomics are beginning to identify additional therapeutic targets beyond cell-surface molecules or cytokines that have traditionally been the targets of drug design. Potential targets of new MS treatments include transcription factors that are important in the development of pathogenic T cells. This approach is already being used in other areas of medicine. For example, PPAR-γ agonists, which are used to treat type 2 diabetes, activate transcription factors to alter glucose metabolism and alleviate hyperglycemia.[45] These same agonists have also been tested in patients with MS because PPAR-γ and PPAR-α agonists also demonstrate anti-inflammatory properties.[46,47]

## Summary and Conclusions

Our collective understanding of the pathogenesis of MS has increased considerably in recent years. A growing body of research has demonstrated how a diverse population of immune cells can participate in inflammatory demyelination and contribute to axon loss. Research has also begun to define mechanisms that can allow for axonal regeneration and remyelination. Conventional neuroimaging techniques are increasingly useful for both diagnosis and treatment of MS, and novel techniques are being developed that identify specific pathological processes within the CNS. The identification of biological markers of MS may help to improve diagnostic accuracy, while also suggesting new targets for therapeutic intervention.
